# Spontaneous rupture of the uterus in the third trimester after high-intensity ultrasound ablation in adenomyosis: A case report

**DOI:** 10.3389/fmed.2022.966620

**Published:** 2022-09-15

**Authors:** Siyun Wu, Jun Liu, Libin Jiang, Lijun Yang, Yanhua Han

**Affiliations:** ^1^Department of Obstetrics and Gynecology, Zhongshan City People's Hospital, Zhongshan, Guangdong, China; ^2^Department of Obstetrics and Gynecology, Zhongshan Torch Development Zone Hospital, Zhongshan, Guangdong, China

**Keywords:** adenomyosis, uterine rupture, third trimester, high-intensity ultrasound ablation, case report

## Abstract

Adenomyosis is a benign uterine disease. Due to the higher incidence of adenomyosis and patients' demands for fertility, high-intensity ultrasound ablation has been widely used in gynecological patients with uterine fibroids and adenomyosis. Ultrasound ablation of lesions can help alleviate symptoms in patients without increasing the incidence of obstetric complications in subsequent pregnancies. High-intensity ultrasound ablation is not considered a risk factor for uterine rupture. However, we describe a case of adenomyosis treated with high-intensity ultrasound ablation presenting with uterine rupture in the third trimester. The patient underwent an emergency cesarean section to deliver the baby successfully and underwent uterine repair surgery. When treating patients with adenomyosis, care should be taken to protect the myometrium, endometrium, and serous layer to reduce the risk of uterine rupture.

## Introduction

Uterine rupture, the rupture of the uterine body or lower segment of the uterus, can occur during late pregnancy and delivery. It is the most severe complication in obstetrics and may endanger the safety of the fetus and mother. The most common risk factors for uterine rupture include a history of uterine surgery, blocked decline due to cord prolapse, and improper use of uterine contraction drugs. Nevertheless, spontaneous uterine rupture also occurs in late pregnancy without associated risk factors. A study published in 2015 reported incidences of ruptured uteri among unscarred and scarred uteri with the results showing 4.54 and 28.60 per 100,000 deliveries, respectively (1).

Adenomyosis is a benign uterine disease characterized by invasion of the endometrial glands and stroma in the myometrium (2). It occurs in women of reproductive age and peaks between 30 and 50 years, and epidemiology shows that the incidence is between 7 and 50% (3).

The incidence of premature birth, uterine rupture, and other obstetric complications with adenomyosis are significantly higher than that in healthy women (4). This is related to the rupture of the myometrium and loss of muscle fibers in muscle cells, resulting in reduced uterine elasticity (5).

The traditional treatment for adenomyosis is hysterectomy, which is often considered the only solution for pain relief. However, due to the higher incidence of adenomyosis and patients' demands for fertility, hysterectomy is not a suitable treatment option for all patients. Several studies have reported various surgical methods, including laparoscopic partial adenomyosis resection and uterine trilobectomy (6). However, the risk of late uterine rupture in postoperative pregnancy is significantly higher (7). Therefore, the development of high-intensity ultrasound ablation (HIFU) offers the hope of preserving fertility.

HIFU has been widely used in gynecological patients with uterine fibroids and adenomyosis. The application of HIFU in the treatment of adenomyosis is well-established, and several studies report that ultrasound ablation can achieve the ablation of lesions and alleviate symptoms in patients without increasing the incidence of obstetric complications in subsequent pregnancies (8, 9).

However, a recent case of adenomyosis in our hospital presented with uterine rupture in the third trimester after HIFU therapy.

## Case description

**Figure 1:**
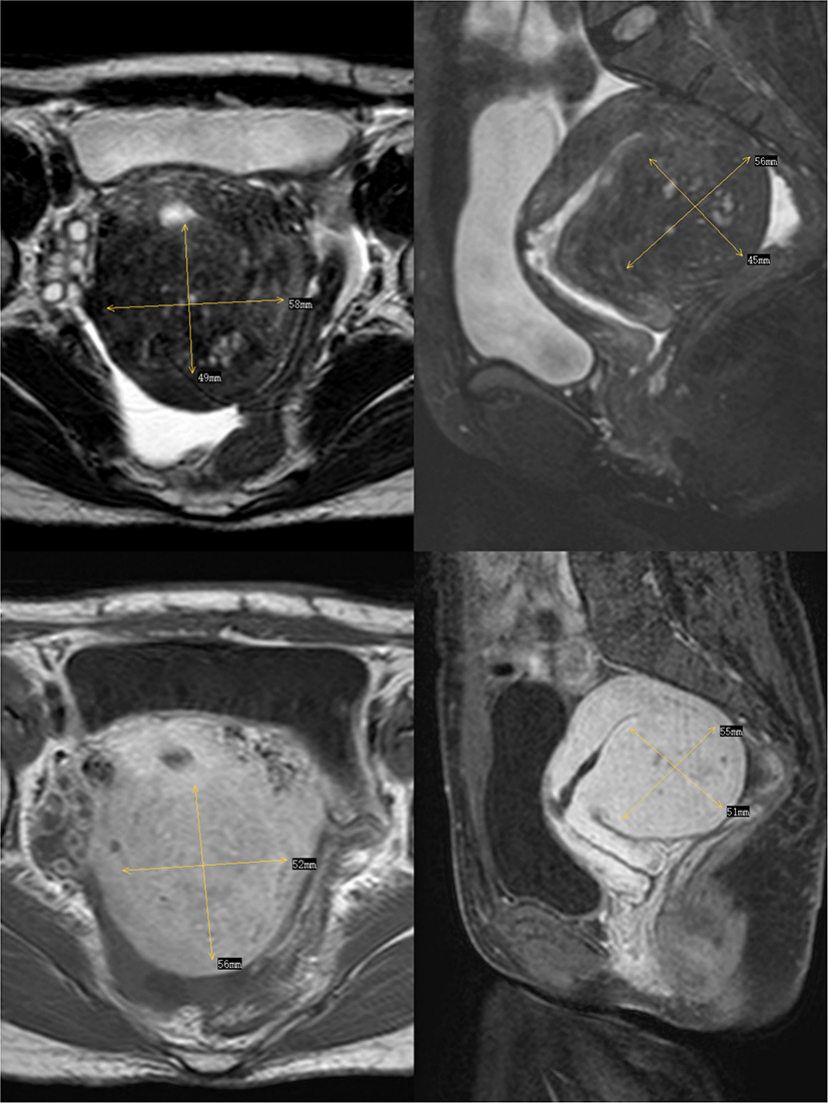
The above two images are T2-weighted images of the transverse and longitudinal sections of the uterus respectively, and the following two images are enhanced images of T1.

**Figure 2:**
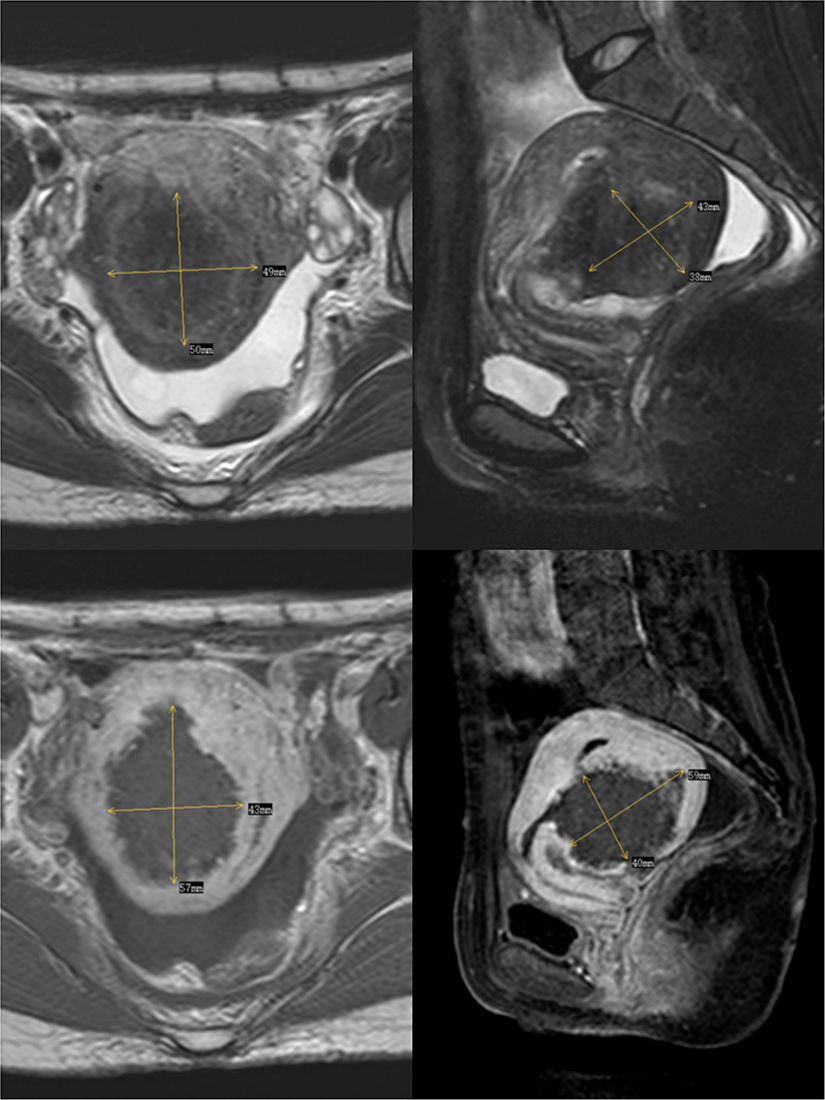
The above two images are T2-weighted images after the ablation of Haifu, while the following two images are enhanced images of T1. The filling defect is where part of the lesions were ablated by Haifu.

**Figure 3:**
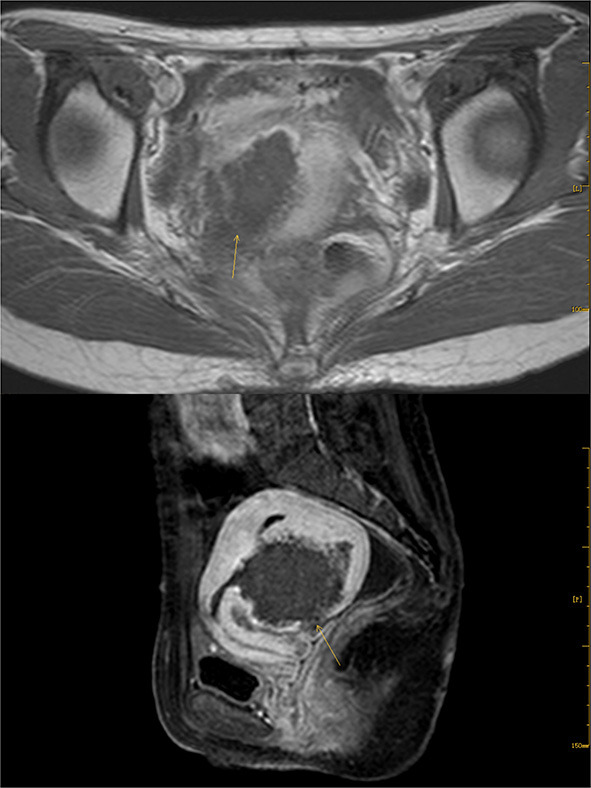
Enhanced image of T1 after Haifu, arrow indicates the filling defect of serosal layer of uterus.

In 2012, the patient delivered a child *via* cesarean section without complications. In 2014, she visited our hospital with dysmenorrhea accompanied by increased menstrual volume. The dysmenorrhea score (Visual Analog Scale/Score, VAS) was seven, and the menstrual volume increased to double the previous menstrual volume. Magnetic resonance (MR) examination in our hospital indicated adenomyosis, which was located in the posterior wall of the uterus, with a size of 58 × 56 × 49 mm (Figure 1). Preoperative blood routine examination, coagulation function, liver and kidney function and electrocardiogram results were normal. Due to the symptoms of increased menstrual volume, the patient underwent endometrial biopsy first. Pathology revealed that the patient did not have endometrial cancer. The patient underwent HIFU at our hospital. The practical energy was 400 W, and the treatment time was 867 s. Postoperative MR (Figure 2) showed that the non-perfused area of the posterior uterine wall lesion was 59 × 40 × 43 mm, and the non-perfused volume ratio was 63.76%. After HIFU therapy, the patient was treated with gonadotropin-releasing hormone for 6 months; the dysmenorrhea score was reduced to one point when the drug was discontinued, and the menstrual volume returned to normal. However, postoperative MR images (Figure 3) showed a filling defect in the serosal layer of the posterior wall of the uterus, suggesting that ablation of the lesion broke through the serosal layer, which may lead to a serosal defect of the uterus. The patient was followed up regularly, and no enlargement of the adenomyosis lesion was found on reexamination.

This, now 34-year-old woman, conceived naturally in July 2020, and regular prenatal examinations showed normal fetal development and no apparent abnormalities in the uterus. On February 03, 2021, at 34 weeks of gestation, the patient presented with sudden dizziness, fatigue, decreased fetal movement, no fainting, nausea, vomiting, abdominal pain, vaginal bleeding, and no vaginal discharge. She was admitted to the hospital.

**Figure 4:**
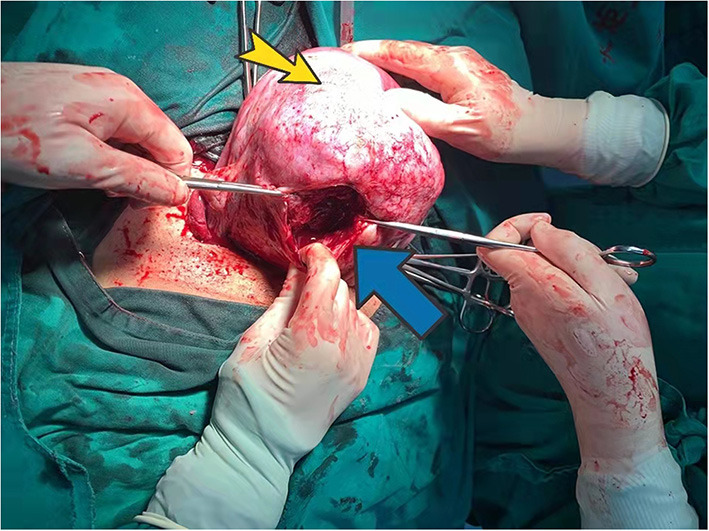
Posterior uterine wall during surgery. Yellow arrow indicates adenomyosis. The blue arrow points to the posterior wall rupture.

Physical examination showed the following: temperature 36°C; pulse rate, 94 bpm; resting heart rate, 25 beats/min; and blood pressure, 85/40 mmHg. We noted abdominal distention, abdominal softness, no tenderness, and no rebound pain. Ultrasonography indicated a single intrauterine pregnancy, the fetus was alive, and the fetal heart rate was low at 70 bpm. Emergency cesarean section was performed, and a baby boy was delivered successfully with an Apgar score of 3-6-6. During the operation, a large amount of blood was found in the pelvic and abdominal cavities (~2,500 ml), and a 12 × 6 cm rupture through the whole uterine wall was observed in the posterior wall (Figure 4). The uterine rupture location was consistent with the breakthrough position of the uterine serosal layer, as suggested by MR after HIFU surgery in 2014. The patient underwent surgery to repair her uterus. A total of 800 ml of concentrated red blood cells were transfused postoperatively. The patient and the neonate recovered well and were discharged 28 days after neonatal treatment.

## Discussion

### Risk factors for uterine rupture

The primary risk factor for uterine rupture is surgical uterine scars. In a report by Osada, the risk of uterine rupture in patients with adenomyosis after surgery was significantly higher than that in patients with uterine fibroids. The risk of uterine rupture was also higher in patients who underwent laparoscopic surgery than in those who underwent open surgery (10). Published reports have shown that in addition to uterine scar formation, there are also risks of uterine malformation or uterine rupture after mediastinal hysteroscopy (11) and Hysteroscopic myomectomy (12). Another study showed that treatment of adenomyosis with HIFU did not increase the risk of uterine rupture (13).

In this case, the patient underwent HIFU, and postoperative MRI angiography suggested a defect in the muscle layer suspicious of a full-thickness infarct, indicating that this part of the muscle layer was necrotic. However, no studies have supported a correlation between muscle layer necrosis and postoperative scar formation. The patient conceived naturally 7 years later, and spontaneous uterine rupture occurred in the late trimester. The location of the uterine rupture was the same as that of the lesions ablated during HIFU surgery, thus, it could be inferred that there was weakness in the muscle layer or scar formation, leading to spontaneous uterine rupture.

### Identification of uterine rupture

Uterine rupture is a high-risk emergency in obstetrics and often endangers the lives of pregnant women and fetuses. Uterine rupture usually occurs after labor has begun and can occur during the first or second stages of labor (14). Clinical signs and symptoms of uterine rupture include sudden onset of lower abdominal pain, with or without peritoneal stimulation; unstable fetal heart rate, such as fetal bradycardia during pregnancy; hypotension; and hemorrhagic shock (15). However, in this case, the patient did not go into labor and had no abdominal pain or contractions. The first symptom was hemorrhagic shock, presenting as dizziness, fatigue, and syncope, accompanied by an abnormal fetal heart rate. Due to the large uterus, a preoperative color ultrasound examination did not reveal any abdominal fluid and the uterine rupture was only observed intraoperatively.

Diagnosis of spontaneous uterine rupture is challenging in the third trimester, however, fetal heart rate abnormalities tend to occur early (16). Therefore, an abnormal fetal heart rate in the third trimester or a full-term pregnancy requires immediate action. Improved auxiliary examinations, such as color ultrasound and MRI, could help exclude acute abdominal diseases, such as appendicitis, calculi, and uterine rupture, to ultimately defer the termination of pregnancy as long as possible (17).

In conclusion, when treating patients with adenomyosis, care should be taken to protect the myometrium, endometrium, and serous layer to reduce the risk of uterine rupture. Further studies are needed to investigate the risk of uterine rupture in patients who underwent previous HIFU and it might be good practice to consider the risk of uterine rupture as a possibility in these patients when providing prenatal care.

### Risk of carcinogenesis in adenomyosis

The common symptoms of adenomyosis are increased menstrual volume and prolonged periods, similar to those of endometrial cancer. Moreover, adenomyosis occurring during perimenopause has a certain risk of malignancy. Therefore, malignant changes should be excluded before conservative treatment (18). In this case, a curettage was performed to exclude endometrial cancer before the HIFU, but the curettage had limitations. Vascular ultrasound examination can be considered to improve the detection rate of endometrial cancer (19).

## Ethics statement

Ethical review and approval was not required for the study on human participants in accordance with the local legislation and institutional requirements. Written informed consent from the [patients/ participants OR patients/participants legal guardian/next of kin] was not required to participate in this study in accordance with the national legislation and the institutional requirements.

## Author contributions

SW was the patient's chief physician and was responsible for the patient's follow-up. This article has been modified by YH. LJ performed a cesarean section on the patient. JL and LY performed the HIFU on the patient. All authors contributed to the article and approved the submitted version.

## Conflict of interest

The authors declare that the research was conducted in the absence of any commercial or financial relationships that could be construed as a potential conflict of interest.

## Publisher's note

All claims expressed in this article are solely those of the authors and do not necessarily represent those of their affiliated organizations, or those of the publisher, the editors and the reviewers. Any product that may be evaluated in this article, or claim that may be made by its manufacturer, is not guaranteed or endorsed by the publisher.
